# Age of the Horse, Ox, Dog, and Other Domesticated Animals

**Published:** 1890-06

**Authors:** R. S. Huidekoper

**Affiliations:** Veterinarian


### AGE OF THE HORSE, OX, DOG, AND OTHER DOMESTICATED ANIMALS.

By R. S. Huidekoper, M.D., Veterinarian.

[*Continued from page* 288.]

### DURATION OF THE LIFE OF THE HORSE.

According to Buffon the duration of the life of the horse is, as in all other species of animals, proportionate to the duration of its growth; man, who is fourteen years in growth, lives six or seven times that length of time; that is to say, he may live to 90 or 100; the horse, which requires about four years to attain its growth, may live six or seven times that length of time, that is, to twenty-five or thirty years. Examples which are contrary to this rule are so rare that they need hardly be regarded as exceptions of consequence; as the more common draft horses acquire their growth in less time than better bred horses ; they also live a shorter time, fifteen or sixteen years.

According to Bourgelat, "the age of the horse can be estimated at eighteen to twenty years. The number of those which pass this age is excessively small."

Aristotle noted that horses which are nourished in stables live for a shorter time than those which are raised in herds ; the conditions of slavery and domesticity diminish their powers of resistance to the wear and tear of life.

Athenæus and Pliny claim to have known the horse to attain the age of sixty-five or even seventy years. Augustus Nipheus speaks of a horse belonging to Ferdinand I, which was over seventy, but these observations are only exceptions, similar to those which we sometimes find in human species.

Hartman and Buffon both note that the life of mares is generally longer than that of horses. This observation was already made by Aristotle (Hist. Animal., lib. V), and corresponds to the same rule in the human race, where women generally live to a greater age than men. Hartman claims that, "it is an undoubtable sign that a breeding horse is well bred and in good health when it is slow in development. Those which do not attain their complete growth until six or seven years, excepting in cases of accident, are useful for twenty years or more, and may be still sound and healthy at thirty years. The other ones, on the contrary, those which attain their growth in four years rarely, pass the age of twenty."

The great heavy draft horses, which attain their growth in even less time, are already aged at ten or twelve years. Examples of horses of thirty, thirty-six, and forty years of age would not be so rare among our animals if the tyranny and hard usage imposed upon them by men did not aid greatly to shorten their lives.

Ordinarily, as soon as the horse has seen its best days, it is sold from the stable in which it has been kept to be replaced by more useful animals, and it goes rapidly into the hands of the hackman and the huckster, doing harder work with less nourishment until it becomes completely used up and depreciates to the value of the knacker's price.

Among the principle causes which modify the longevity of the horse may be counted : the length of time of development, the size of the horse, the work to which the animal is put, and the care which it receives.

We find certain races and certain individuals which are precocious in their development, and other races and sub-divisions of families in which longevity is hereditary.

M. Bouley says : " There are tardy races and precocious races. In the last the precocity is the result of the combined influence of heredity and alimentation, in which the organic formation acts in a precipitous way, as it were, in those individuals which compose it, and we find a hasty achievement of development ; from which it results, that the duration of their first part of life is so much shortened, and as a fatal consequence also that of their whole life, for this more rapid development, impressed on their organisms has no other termination from the industrial point of view, which produces it, than to hasten the moment of death."

The small races of horses and small individuals last usually for a longer time than larger ones. We can make no satisfactory explanation of this, unless it is that in the larger animals the wear and tear is greater from the very fact of the animal having so much more to take care of in itself. The character of work, to which the horse is put, is an important factor in shortening the life, due to the fatigue entailed and the using up of the powers of the organs. We find that a horse with a quiet and docile disposition will perform heavier and rougher work, with less deterioration in its animal economy, than one alongside of it of a nervous and excitable disposition. The more calm and quiet life of horses in the country is less prejudicial to their existence than the bustle and confusion of the great industrial centres and crowded streets of our large cities.

Again, the necessities of rapid and variable work and irregular hours of feeding in great cities predispose to internal diseases, while the greater loads and rough pavements lead to strains of tendons, wrenches of bones, bruised feet and other injuries which soon render the animals unserviceable or condemn them to return to the softer ground, lighter work and more regular life of the country, with, however, a broken-down constitution. The care which the horse receives from its keeper — which, of course, includes the regulation of work and feed—is an all-important matter. A kind, judicious owner will work horses for years and keep them sound and in good condition, doing a vast amount of work, while in the next stable, under the guidance of a careless or brutal owner, equally good horses will perform less work and become worthless in a short time.

The word "*used*," applied to horses, has become synonymous with the word "*aged*" applied to man, and indicates the time when the animal has become prematurely old.

Examples are not rare in which the horse has attained the age of 30, 36, 40 and even more years, and has been in perfect health and capable of moderate useful service.

### PRINCIPLES OF EXAMINATION FOR DETERMINING THE AGE OF THE HORSE.

To anyone accustomed to horses it is an easy matter to distinguish at sight the very young from the adult horse, and the middle aged from the very old animal. In very old animals white hairs commence to show in the neighborhood of the temples and around the eyes and the nostrils, if the color of the animals is dark; gray and roan horses become very much lighter in color, and even at times nearly white; the inferior extremity of the head becomes pointed, and the sides of the face become depressed; there is an evident change in the line of the back and loins, which become depressed (sway backed). The aplomb of the legs and the blemishes found on them are common witnesses of work which has lasted for a number of years.

In addition to the examination of the teeth, which is frequently considered the only point that need be looked at to determine age, there are many other points which are of very great value.

We have seen that the molar teeth of the very young animal are deeply imbedded in the alveolar cavities of the jawbone, and that when the animal becomes older the teeth are gradually pushed out from these cavities, as the table of the tooth becomes worn away. The molars are pushed out and the jaws become thinner by absorption of the bone, which is no longer needed as a bed for holding the teeth, which are diminished in size. We find, then, that, as the horse becomes gradually older, the branches of the jawbone, which in the young animal were thick and rounded, become gradually thinner and sharp on their border, and form a very accurate indication of the approximate age of the animal.

In an old Arab book of agriculture, "Ibn-el-Awamm," written about the Twelfth Century, it is stated that "One of the signs by which an old horse can be recognized is to pinch between the thumb and index finger the skin of the forehead and draw it out, and then to let go of it quickly. If the skin returns promptly to its place and becomes perfectly smooth, the animal will make a good horse," etc.

In Aristotle we find the same statement prescribed as an indication of age; "if the skin returns promptly to its place and leaves no wrinkle, the animal is young; if the skin remains wrinkled, it is old."

An old and neglected, but useful means, of approximating the age of old horses is by means of the "knots" in the tail. These knots are little prominent eminences on either side of the base of the tail, formed by the transverse process of the coccygeal bones. The processes can be felt in young horses, and become especially prominent after the emaciation of a severe illness, but in this case they are rounded and are apparently continuous with the other tissues, while in old horses, due to absorption in the bone itself, they become more distinct and seem to stand out from the muscles and softer structures of the tail.

The knots are felt distinctly at the base of the tail when the horse has attained the age of 13 years. In two years later, when they have become more prominent, they have behind them a distinct little depression two or three lines in width. At 16 a second pair of knots are fonnd, which, like the first, in about two years, have behind them a distinct depression, and so on, every three years, a new pair of knots furnish an approximately accurate indication of the age of the animal.

The teeth, however, are by far the most important witnesses of the age of the horse. The examination of these, which includes the incisors, the tusks and the molars, shows: *a.* If the incisive arch is composed of teeth of the first or second dentition, or of both; *b.* If there is a normal number of them; *c.* The situation, length, breadth and the angle which they have in the maxillary bones; *d.* If the tables of the teeth touch each other, or if one overreaches the other, which somewhat modifies the leveling of the table; *e.* The color and substances on the face of the teeth; *f.* If they have been subjected to fraudulent handling.

In the incisors we study the form and details of the table, the direction and length of the tooth, and the condition of the corner tooth. In the tusks, we note the amount of use to which the teeth have been subjected, their direction and their length. In the molars we look for the number and the dentition to which they belong, the condition of their grinding surface, their length and direction and the integrity of their substance, and that of the gums which surround them.

### CHARACTERS FURNISHED BY THE TEETH.

The reader may, perhaps, have wondered why we should have gone into such minute detail, in the anatomical description of the teeth, but an accurate knowledge of all these details is absolutely necessary, when we take into consideration the slow, insensible and variable wearing, producing often only trivial changes, which are, however, important guides.

Washed incessantly, rubbed, used and broken as the teeth are by the action of the saliva, the lips, the cheeks, the tongue and the food which the animal takes, we have to take into consideration every detail of structure and position of them, the breed and general condition of the animal, and the surroundings in which it has been raised. A thoroughbred with dense bones and hard teeth, will wear the latter away much more slowly than a coarse-boned lymphatic common horse with softer substances in the teeth. The character of food to which a colt has been accustomed will stimulate or diminish the functional activity of the tooth, and, while hard substances would naturally wear a tooth faster than softer food, yet, the animal which has been raised on the former will have harder teeth than one which has not had to use them so severely. Any one who has only been accustomed to examine the mouths of horses of one section of country will find that he must extend his ideas and adapt himself to a new condition of things, when called upon to judge of the age of horses from another region. All horses, except thoroughbreds, are supposed to take their age from May 1st; the latter count their years from January 1st.

If a horse's mouth presents exactly the characters which indicate a certain number of years of growth, we say that it "is — years;" if it has not quite attained the age, it is described as "rising — years;" if it has passed the period and has not yet attained the markings of another year, it is counted as "— years off."

The natural division of the two periods of age, as indicated by the temporary and the permanent teeth, is subdivided as follows :

1. The period of eruption of the incisors of first dentition.
2. The leveling of these teeth and their progressive use.
3. The period of the falling out of the deciduous teeth and the appearance of the permanent ones.
4. The leveling of these latter.
5. The successive forms which their tables present as the teeth become worn away.

[TO BE CONTINUED.]

